# Basic Apoptotic Mechanisms of Lead Toxicity in Human Leukemia (Hl-60) Cells

**DOI:** 10.3390/ijerph7052008

**Published:** 2010-04-28

**Authors:** Clement G. Yedjou, Jessica N. Milner, Carolyn B. Howard, Paul B. Tchounwou

**Affiliations:** Cellomics and Toxicogenomics Research Laboratory, NIH-Center for Environmental Health, College of Science, Engineering and Technology, Jackson State University, 1400 Lynch Street, P.O. Box 18540, Jackson, MS 39217, USA; E-Mails: jessica.n.milner@jsums.edu (J.N.M.); carolyn.b.howard@jsums.edu (C.B.H.); paul.b.tchounwou@jsums.edu (P.B.T.)

**Keywords:** lead nitrate, HL-60 cells, annexin V, caspase-3, flow cytometry, apoptosis

## Abstract

Lead exposure represents a medical and public health emergency, especially in children consuming high amounts of lead-contaminated flake paints. It may also cause hematological effects to people of all ages. Recent studies in our laboratory have indicated that apoptosis may be associated with the lead-induced oxidative stress and DNA damage. However, the mechanisms underlying its effect on lymphocytes are still largely unknown. Therefore, the aim of the present study was to investigate the apoptotic mechanisms of lead nitrate [Pb(NO_3_)_2_] using HL-60 cells as a test model. HL-60 cells were treated with different concentrations of Pb(NO_3_)_2_ for 24 h prior to cell viability assay and flow cytometry assessment. The results obtained from the trypan blue exclusion test indicated that at very low concentration, Pb(NO_3_)_2_ has no effect on the viability of HL-60 cells. A significant (*p < 0.05*) decrease in cell viability was observed when exposed to high level of Pb(NO_3_)_2_. Data generated from the flow cytometric assessment indicated that Pb(NO_3_)_2_ exposure significantly (*p < 0.05*) increased the proportion of annexin V positive cells (apoptotic cells) compared to the control. Pb(NO_3_)_2_ induced apoptosis of HL-60 cells was associated with the activation of caspase-3. In summary, these studies demonstrated that Pb(NO_3_)_2_ represents an apoptosis-inducing agent in HL-60 promyelocytic leukemia cells and its apoptotic mechanism functions, at least in part via, induction of phosphatidylserine externalization and caspase-3 activation.

## 1. Introduction

Lead serves no useful purpose in the human body, and its presence in the body can lead to toxic effects, regardless of exposure pathway. It is classified as a Group B2 carcinogen (possible human carcinogen) by the International Agency for Research on Cancer. Over the past decade, it has become increasingly evident that contact with lead, even at very low levels, can produce serious adverse health effects, especially in young children [1,2]. One of the primary targets for lead is the nervous system. Bressler and his collaborators reported that lead exposure causes neurotoxic effects such as behavioral abnormalities, learning impairment, decreased hearing, and impaired cognitive functions in human and experimental animals [3]. However, the mechanisms underlying the effects of lead on lymphocytes remain largely unknown. Recent studies in our laboratory indicated that apoptosis might be associated with lead-induced oxidative stress and DNA damage in cancer cells [4,5].

Apoptosis is a form of cell death in which a programmed sequence of events leads to the elimination of cells without releasing harmful substances into the surrounding area. It plays a crucial role in developing and maintaining health by eliminating old cells, unnecessary cells, and unhealthy cells. The human body replaces perhaps a million cells a second. The regulation of apoptosis is complex and two important pathways are involved. In extrinsic apoptosis, external signals interact with death receptors, which in turn activate caspase-8 and caspase-3/-7. In intrinsic apoptosis, the loss of mitochondrial transmembrane potential may initiate cytochrome *c* release, which binds to apoptotic activating factor-1 (Apaf-1) and activates caspase cascade.

Several scientific studies using rat model have revealed that lead induces apoptosis in many experimental systems, including rat brain [6,7], rat testis [8], rat fibroblasts [9], rat lung [10], and rat and mouse retinal rod cells [11,12]. Although the apoptotic potential of lead is well documented, the mechanism underlying its effect on lymphocytes is still largely unknown. Therefore, the present study was designed to investigate the molecular mechanisms of lead nitrate [Pb(NO_3_)_2_] toxicity using HL-60 cells as an *in vitro* test model and to determine whether phosphatidylserine externalization and active caspase-3 (an important executioner of apoptosis) are involved in lead toxicity.

## 2. Material and Methods

### 2.1. Chemicals and Media

Reference solution (1000 ± 10 ppm) of lead nitrate [Pb(NO_3_)_2_] (CAS No. 10099-74-8, Lot No. 981735-24) with a purity of 100% was purchased from Fisher Scientific in Fair Lawn, New Jersey. Growth medium RMPI 1640 containing 1 mmol/L L-glutamine was purchased from Gibco BRL products (Grand Island, NY). Fetal bovine serum (FBS), phosphate buffered saline (PBS), and trypan blue exclusion kit were obtained from Sigma Chemical Company (St. Louis, MO). Annexin V/PI staining and active caspase-3 kits were purchased from BD Biosciences (Pharmingen, CA).

### 2.2. Cell/Tissue Culture

The HL-60 promyelocytic leukemia cell line was purchased from the American Type Culture Collection—ATCC (Manassas, VA). It is a promyelocytic cell line derived from peripheral blood cells from a 36 year-old Caucasian female with acute promyelocytic leukemia (APL). In the laboratory, HL-60 cells were stored in the liquid nitrogen until use. They were thawed by gentle agitation of their containers (vials) for 2 min in a water bath at 37 °C. After thawing, the content of each vial of cells was transferred to a 25 cm^2^ tissue culture flask, diluted with up to 10mL of RMPI 1640 containing 1 mmol/L L-glutamine (GIBCO/BRL, Gaithersburg, MD) and supplemented with 10% (v/v) fetal bovine serum (FBS), and 1% (w/v) penicillin/streptomycin. The 25cm^2^ culture flasks containing 2 × 10^6^ viable cells were observed under the inverted microscope, followed by incubation in a humidified 5% CO_2_ incubator at 37 °C. Three times a week, they were diluted and maintained under same conditions at a density of 5 × 10^5^ cells/mL and harvested in the exponential phase of growth. The cell viability was assessed by the trypan blue exclusion test (Life Technologies) and manually counted using a hemocytometer.

### 2.3. Cell Treatment and Measurement of Cell Viability

To 180 μL aliquots in six replicates of the cell suspension (5 × 10^5^ cells/mL) seeded to 96-well polystyrene tissue culture plates, 20 μL aliquots of stock solutions of lead nitrate [Pb(NO_3_)_2_] were added to each well using distilled water as solvent to make-up final concentrations of 0.78, 1.56, 3.12, 6.25, 12.50, 25.00, and 50.00 μg/mL of Pb(NO_3_)_2_, respectively. Control cells received 20μL of distilled water. All chemical exposures were carried in 96-well tissue culture plates for the purpose of chemical dilutions. Cells were placed in a humidified 5% CO_2_ incubator for 24 h at 37 °C. After incubation, the cell viability was assessed by the trypan blue exclusion test (Life Technologies) using a hemocytometer to manually count the cells. Briefly, ten μL of a 0.5% solution of the dye was added to 100 μL of treated cells (1.0 × 10^5^ cells/mL). The suspension was then applied to a hemocytometer. Both viable (transparent) and nonviable (blue) cells were counted. A minimum of 200 cells were counted for each data point in a total of eight microscopic fields.

### 2.4. Annexin V FITC/PI Binding Assay by Flow Cytometry

The response of HL-60 cells to lead nitrate [Pb(NO_3_)_2_] was assessed by flow cytometry using Annexin V FITC/PI staining kit. After 24 h of exposure to various concentrations Pb(NO_3_)_2_, 1 × 10^6^ cells/mL were washed in PBS, re-suspended in binding buffer (10mM Hepes/NaOH pH 7.4, 140 mM NaCl, 2.5 mM CaCl_2_), and stained with FITC-conjugated annexin V (Pharmingen, Becton Dickinson Co., San Diego, CA, USA). Then, cells were incubated for 15 min in the dark at room temperature, washed with binding buffer and analysed by flow cytometry (FACS Calibar; Becton-Dickinson) using CellQuest software.

### 2.5. Active Caspase-3 Assay by Flow Cytometry

Caspase-3 assay was carried out using a commercially available kit (Phycoerythrin-Conjugated Polyclonal Active Caspase-3 Antibody Apoptosis Kit, Pharmingen). Control and Pb(NO_3_)_2_-treated cells were assayed for caspase-3-like protease according to the manufacturer’s instructions. Briefly, 1 × 10^6^ cells/mL were washed per concentration with cold PBS (pH 7.4). Washed cells were suspended in Cytofix/Cytoperm solutions and incubated for 20 min on ice. Cells were pelleted and washed with Perm/Wash buffer. Cells were then centrifuged and re-suspended in 0.2 mL Perm/Wash, 20 μL PE-conjugaled polyclonal rabbit anti-active caspase-3 antibody and incubated at room temperature for 30 min. Cells were washed in 1mL Perm/Wash buffer and then re-suspended in 0.5 mL of the same buffer prior to analysis by flow cytometry (FACS Calibar; Becton-Dickinson) using CellQuest software.

### 2.6. Statistical Analysis

Data were presented as means ± SDs. Statistical analysis was done using one way analysis of variance (ANOVA Dunnett’s test) for multiple samples. Student’s paired test was used to analyze the difference between the controls and lead nitrate-treated cells. All p-values < 0.05 were considered to be significant. Graphs were made to illustrate the dose-response relationship with respect to cell viability. Tables were constructed to illustrate the dose-response relationship with respect to annexin V and caspase-3 negative and positive cells.

## 3. Results and Discussion

### 3.1. Cell Viability

**Figure 1.**
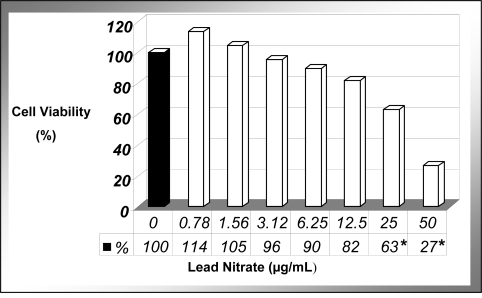
Cytotoxic effect of Pb(NO_3_)_2_ to HL-60 cells. HL-60 cells were cultured in the absence or presence of Pb(NO_3_)_2_ for 24 h as indicated in the Materials and Methods. Cell viability was determined based on the trypan blue exclusion test. Each point represents a mean value of 3 experiments with 6 replicates per dose. *P < 0.05 versus compared with control group.

The present study demonstrated that at the cellular level, lead nitrate [Pb(NO_3_)_2_] significantly (P < 0.05) reduced the viability of human leukemia (HL-60) cells in a dose-dependent manner (Figure 1). As shown in this figure, there was a slight increase in cell viability at 0.78 μg/mL compared to the control, but this slight increase was not statistically significant. Between 1.56 and 3.78 μg/mL, the viability was similar to the control cells. At 6.25μg/mL and higher concentration of Pb(NO_3_)_2_, the viability of HL-60 cells decreased significantly and reached a statistical significance (*P < 0.05*) between 25 and 50 μg/mL. The chemical dose (LD_50_) required to kill 50% of the cell population was computed to be 34.14 + 8.51 μg/mL upon 24 h of exposure. These results are consistent with those of previous investigations reporting a marked reduction in the viability of cancer cells following exposure to lead [13,14]. We previously reported a similar trend with arsenic trioxide-treated HepG_2_ cells in our laboratory [15]. A recent study indicates that lead is highly toxic to immune cells by inhibiting cell adhesion property, and altering cell morphology in the splenic macrophages of mice [16].

Although fatal lead poisoning occurs rarely in the United States, several epidemiological studies have pointed out that it represents a medical and public health emergency, especially in children consuming high amounts of lead-contaminated flake paints [17]. Death in these lead-poisoned children has been associated with extreme lethargy with facial palsy and gasping respirations consistent with lead encephalopathy, and severe hematologic abnormalities [17]. Based on the result of trypan blue exclusion test, Pb(NO_3_)_2_ at concentrations of 10, 20, 30 and 40 μg/mL were used in the subsequent apoptosis-related experiments.

### 3.2. Induction of Phosphatidylserine Externalization

**Figure 2.**
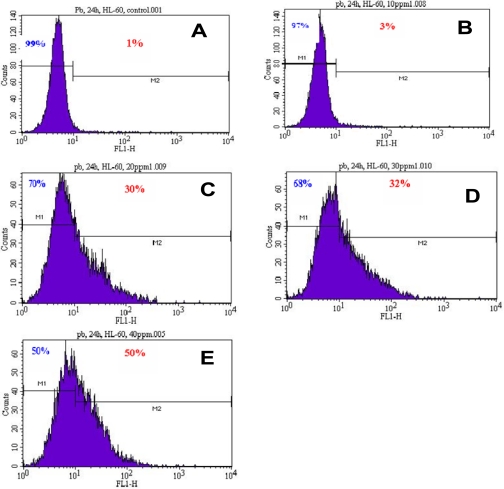
Representative histograms showing a comparison of the distribution of negative annexin V cells (M1) and positive annexin V cells (M2) after 24 h incubation of lead nitrate in HL-60 cells. A, control; B, 10 μg/mL Pb(NO_3_)_2_; C, 20 μg/mL Pb(NO_3_)_2_; D, 30 μg/mL Pb(NO_3_)_2_; E, 40 μg/mL Pb(NO_3_)_2_.

To gain insight into the mechanism of lead-induced apoptosis of HL-60 cells, we examined the possible involvement of phosphatidylserine externalization. As seen in figure 2, there is a gradual increase in annexin V positive cells (apoptotic cells) with increasing concentrations of Pb(NO_3_)_2_ in HL-60 cells. We found that Pb(NO_3_)_2_ exposure significantly increased the percentage of annexin positive cells (apoptotic cells) compared to the control cells in a dose-dependent manner. Upon 24 h of Pb(NO_3_)_2_ exposure, the proportion of annexin V positive cells (apoptotic cells) revealed by the flow cytometry analysis were 1.00 ± 0.00%, 3.00 ± 0.28%, 33.54 ± 5.15%, 38.50 ± 8.56%, and 48.15 ± 23.04% in 0, 10, 20, 30, and 40 μg/mL, respectively (Table 1). These results suggest that Pb(NO_3_)_2_ is a potent inducer of apoptosis. Lead nitrate effect was more powerful at the highest dose (40 μg/mL) tested with almost 50% of apoptotic cells compared to the control sample with only 1% of apoptotic cell. Annexin-V is a specific phosphatidylserine-binding protein used to detect apoptotic cells by providing an assessment of the progression from living cells (annexin−/PI−) towards apoptotic stage (annexin+/PI−) and postapoptotic cell death (annexin+/PI+).

### 3.3. Activation of Caspase-3

**Figure 3.**
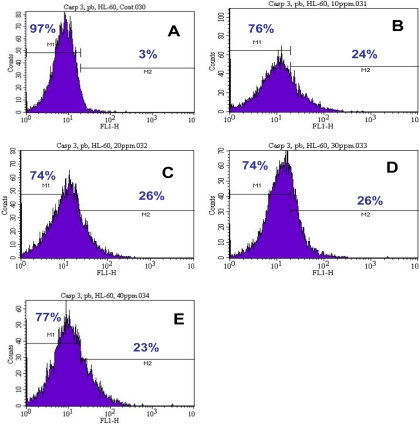
Representative histograms showing a comparison of the distribution of negative caspase-3 cells (M1) and positive caspase-3 cells (M2) after 24 h incubation of lead nitrate in HL-60 cells. A, control; B, 10 μg/mL Pb(NO_3_)_2_; C, 20 μg/mL Pb(NO_3_)_2_; D, 30 μg/mL Pb(NO_3_)_2_; E, 40 μg/mL Pb(NO_3_)_2_.

To further gain insight into the mechanism of lead-induced apoptosis of HL-60 cells, we also examined the possible involvement of caspase-3 activation. Upon 24 h of exposure, the percentages of caspase-3 positive cells (apoptotic cells) were 3.00 ± 0.00%, 30.75 ± 10.25%, 36.50 ± 14.84%, 32.25 ± 8.13%, and 32.50 ± 13.57% in 0, 10, 20, 30, and 40μg/mL of lead nitrate [Pb(NO_3_)_2_], respectively (Table 2). Our results confirmed that Pb(NO_3_)_2_ induced apoptosis of HL-60 cells is associated with the activation of caspase-3 (Figure 3). Caspase-3 is the most frequently activated protease in mammalian cell apoptosis. At 30 and 40μg/mL Pb(NO_3_)_2_ exposure, HL-60 cells failed to undergo further apoptosis, probably due to high level of cell death at these concentrations tested. It is activated through limited proteolysis within its interdomain linker by an initiator caspases, and occasionally by other proteases under specific circumstances [18]. In multiple cell types, activated caspase-3 will result in certain apoptotic hallmarks, such as chromatin condensation and DNA fragmentation [19]. Additionally, caspase-3 plays an important role in the downstream of mitochondrial pathway, after dysfunction of mitochondria and release of cytochrome *c*. Scientific reports have indicated that low to moderate level of lead exposure produces apoptotic rod and bipolar cell death in developing and adult rats [20] and apoptotic neuronal cell death in primary cultured cells [21].

## 4. Conclusions

The present study suggests that lead nitrate [Pb(NO_3_)_2_] exposure lead to the activation of several cellular and molecular processes including, induction of cell death, externalization of phosphatidylserine, and activation of caspase-3 in human leukemia (HL-60) cells. Although the molecular mechanism of lead-induced apoptosis in cancer cells remains largely unclear, we demonstrate that Pb(NO_3_)_2_-induced toxicity in human leukemia (HL-60) cells is associated with apoptosis.

## Figures and Tables

**Table 1.** Summary data of annexin V assay obtained from the flow cytometric. HL-60 cells were cultured in the absence or presence of Pb(NO_3_)_2_ for 24 h as indicated in the Materials and Methods. Values are shown as mean ± SD value of 3 experiments with 6 replicates per dose.

**Concentrations**	**Annexin V Negative Cells or Viable Cells (Mean ± SD)%**	**Annexin V Positive Cells or Apoptotic Cells (Mean ± SD)%**
0μg/mL	99.00 ± 0.00	1.00 ± 0.00
10μg/mL	97.00 ± 0.28	3.00 ± 0.28
20μg/mL	66.46 ± 5.15*	33.54 ± 5.15*
30μg/mL	61.50 ± 8.56*	38.50 ± 8.56*
40μg/mL	51.85 ± 3.04*	48.15 ± 3.04*

* *P* < 0.05 *versus* compared with control group.

**Table 2.** Summary data of caspase-3 assay obtained from the flow cytometry. HL-60 cells were cultured in the absence or presence of Pb(NO_3_)_2_ for 24 h as indicated in the Materials and Methods. Values are shown as mean ± SD value of 3 experiments with 6 replicates per dose.

**Concentrations**	**Caspase-3 Negative Cells or Viable Cells (Mean ± SD)%**	**Caspase-3 Positive Cells or Apoptotic Cells (Mean ± SD)%**
0 μg/mL	97.00 ± 0.00	3.00 ± 0.00
10 μg/mL	69.25 ± 10.25*	30.75 ± 10.25*
20 μg/mL	63.50 ± 14.84*	36.5 0 ± 14.84*
30 μg/mL	67.75 ± 8.13*	32.25 ± 8.13*
40 μg/mL	67.50 ± 13.57*	32.50 ± 13.57*

* *P* < 0.05 *versus* compared with control group.
